# Short-term 30-day adverse events following awake versus asleep deep brain stimulation for movement disorders: a nationwide registry-based study

**DOI:** 10.1016/j.bas.2025.104393

**Published:** 2025-08-13

**Authors:** Victor Gabriel El-Hajj, Ryan Nguyen, Abdul Karim Ghaith, Victor E. Staartjes, Christian Möhrlen, Adrian Elmi-Terander, Rushna Ali

**Affiliations:** a Department of Clinical Neuroscience, Karolinska Institutet, Stockholm, Sweden; b Department of Neurological Surgery, Mayo Clinic, Rochester, MN, USA; c Department of Neurological Surgery, Johns Hopkins University, Baltimore, MD, USA; d Machine Intelligence in Clinical Neuroscience & Microsurgical Neuroanatomy (MICN) Laboratory, Department of Neurosurgery, Clinical Neuroscience Center, University Hospital Zurich, University of Zurich, Zurich, Switzerland; e Department of Anesthesiology and Perioperative Care, University Hospital Zurich, Zurich, Switzerland

**Keywords:** Deep brain stimulation, Awake, Asleep, General anesthesia, Local anesthesia

## Abstract

**Introduction:**

Deep Brain Stimulation (DBS) is FDA-approved for the management of medically refractory movement disorders and epilepsy. We aim to assess potential differences in adverse eventsamong patients undergoing asleep versus awake DBS, to facilitate a patient centric decision-making process for the selection of ideal anesthesia modality for individuals undergoing DBS procedures.

**Methods:**

The ACS National Surgical Quality Improvement Program (NSQIP) database was queried for all patients undergoing DBS treatment between 2011 and 2020 in patients with a diagnosis of Parkinson's Disease, and Essential Tremor. Propensity score matching in a 2:1 ratio was performed. The primary endpoint was to quantify any short-term adverse events.

**Results:**

In total, 1778 patients undergoing asleep (75.7 %) and awake DBS procedures (24.3 %) were identified. The median age among included was 68.0 with most being males (65 %). After 2:1 propensity score matching there was no remaining baseline difference. 30-day complication rates were comparable between groups (2.3 % asleep vs. 0.7 % awake; p = 0.062). Similarly, there were no significant differences in 30-day readmission (3.5 % vs. 3.5 %; p = 0.96), reoperation (1.4 % vs. 0.9 %; p = 0.48), or non-home discharge (3.5 % vs. 3.0 %; p = 0.63). Median hospital length of stay did not differ significantly (0 vs. 0 days; p = 0.23).

**Conclusion:**

In this matched analysis using data from a prospective multicenter database of U.S. hospitals, asleep and awake DBS demonstrated comparable 30-day outcomes. No significant differences were observed in complication rates, readmissions, reoperations, discharge disposition, or length of hospital stay. These findings support clinical equipoise between the two approaches and underscore the importance of tailoring the choice of technique to individual patient characteristics and preferences.

## Highlights

• There was no significant difference in 30-day adverse events between awake and asleep DBS.
• Readmission rates and length of hospital stay were also similar among anesthesia types.
• Our findings support clinical equipoise, aligning with the literature.
• The choice of anesthesia should be individualized based on patient-specific factors.

## 1 Introduction

Deep brain stimulation (DBS) is a safe and effective neuromodulation technique used to treat various neurological conditions (Ghaith et al., 2024a). DBS has been heavily studied in the treatment of movement disorders, receiving FDA approval for use in essential tremor, dystonia, and Parkinson's disease (PD) (Hacker et al., 2021). Other indications include epilepsy and obsessive-compulsive disorder, while several others are the subject of ongoing investigation (Lozano et al., 2019; Peltola et al., 2023; Harmsen et al., 2020). The traditional approach to DBS surgery involves the awake placement of intracerebral electrodes under local anesthesia and intermittent IV sedation (LA) (Krauss et al., 2021). This is often performed alongside microelectrode recording (MER) or other neuromonitoring techniques meant to improve the accuracy of electrode placement. However, advancements in intraoperative imaging have facilitated the use of general anesthesia (GA) to perform asleep DBS surgeries with or without MER, potentially reducing operative time, risk of intracranial hemorrhage due to single pass, and cognitive decline associated with fewer penetrations through the frontal lobe (Kochanski and Sani, 2018). This option is useful for patients who are claustrophobic, or those with psychiatric or even physical limitations that make them unsuitable for awake surgery.

Comparisons between general and non-general anesthesia modalities are not new in the field of neurosurgery (Ghaith et al., 2024b; El-Hajj et al., 2024) and have even been the subject of several previous studies addressing DBS surgery (Iu et al., 2019; Stenmark et al., 2024). These studies have mainly addressed long-term clinical outcomes. Two randomized clinical trials comparing awake and asleep DBS for Parkinson's disease were recently published. Both studies observed no significant difference in motor or cognitive outcomes between the techniques (Holewijn et al., 2021; Engelhardt et al., 2021). Currently, the selection of anesthesia modality during DBS placement is primarily influenced by institutional resources, anesthesiologic risk assessment, and surgeon as well as patient preference (Lee et al., 2022), informed by a comprehensive understanding of the risks and benefits associated with both asleep and awake techniques (Tsai et al., 2019). However, short-term postoperative adverse events following awake versus asleep DBS remain understudied. This is particularly relevant, as the potential benefits of local anesthesia on the risks of immediate perioperative adverse events and lengths of hospital stay may be relevantly different for DBS (Chen et al., 2017) than for other neurosurgical procedures (Rajjoub et al., 2024). Additionally, although effectiveness outcomes have consistently been comparable between the two techniques, we have yet to determine if there are pre-existing patient-specific characteristics that make certain patients more suitable for one versus the other.

The goal of the current study is to determine adverse event occurrences among patients undergoing asleep versus awake DBS, in an attempt to facilitate an evidence based decision-making process on the optimal approach.

## 2 Methods

### 2.1 Database and study population

The American College of Surgeons’ National Surgical Quality Improvement Program (ACS-NSQIP) includes prospective patient data from over 600 participating hospitals across the United States. The database contains only data from the United States. This database facilitates the study of surgical techniques through the collection of detailed patient characteristics and postoperative outcomes and adverse events.

The ACS-NSQIP registry 2011–2020 was queried to identify all patients between receiving DBS treatment for Parkinson's disease and essential tremor. Patients undergoing DBS for dystonia were excluded as the sample did not reach satisfactory size. This step was performed using the following Current Procedural Terminology (CPT) codes 61860, 61880, 61885, 61886, 61888, 61863, and 61867. This study observed the Strengthening the Reporting of Observational Studies in Epidemiology (STROBE) guidelines for cohort studies. The study was considered exempt from Institutional Review Board (IRB) approval and did not require patient consent as all data was de-identified prior to registry inclusion, as per the Mayo IRB.

### 2.2 Primary endpoint

Adverse events were defined as any of the following events including extended length of hospital stays (>7 days), any complication, readmission, reoperation, or mortality within 30-days following the procedure. Complications included superficial skin infection, deep infection, postoperative pneumonia, unplanned intubation, pulmonary embolism, acute renal failure, urinary tract infection, sepsis, myocardial infarction (MI), and ischemic or hemorrhagic stroke. Only 30-day adverse events were available in the registry.

### 2.3 Independent variables

Variables included a range of baseline parameters including information on demographics as well as comorbidities, BMI, smoking, and functional status. Table 1 lists the complete dataset. Lab values were also available and were classified as follow: elevated creatinine (>1.3 mg/dL), elevated BUN (>24 mg/dL), hyponatremia (<135 mEq/L), elevated white blood count (WBC) (>11,000), anemia (hematocrit <41 % for males and <36 % for females), and low platelet count (<150, 000). Obesity was considered when patients had a BMI >30.

**Table 1:** Baseline characteristics prior to propensity-score matching.

Characteristic	Overall, N = 1778	General, N = 1346	Local, N = 432	p-value
**Age**	68.0 (61.0, 73.0)	67.0 (61.0, 73.0)	68.0 (61.0, 74.0)	**0.041**
**BMI**	27.1 (24.0, 31.0)	27.1 (23.9, 31.0)	27.3 (24.5, 31.0)	0.55
**Male sex**	1162 (65 %)	878 (65 %)	284 (66 %)	0.85
**Race**				0.80
White	1698 (96 %)	1284 (95 %)	414 (96 %)	
Black	31 (1.7 %)	23 (1.7 %)	8 (1.9 %)	
Asian	49 (2.8 %)	39 (2.9 %)	10 (2.3 %)	
**Hispanic ethnicity**	105 (5.9 %)	74 (5.5 %)	31 (7.2 %)	0.20
**Diagnosis**				**0.021**
Essential tremor	475 (27 %)	378 (28 %)	97 (22 %)	
Parkinson's disease	1303 (73 %)	968 (72 %)	335 (78 %)	
**In- vs. outpatient procedure**				**<0.001**
Inpatient	464 (26 %)	286 (21 %)	178 (41 %)	
Outpatient	1314 (74 %)	1060 (79 %)	254 (59 %)	
**Transferred from**				0.53
Acute care hospital inpatient	7 (0.4 %)	6 (0.4 %)	1 (0.2 %)	
Nursing home - Chronic care - Intermediate care	22 (1.2 %)	17 (1.3 %)	5 (1.2 %)	
Outside emergency department	1 (<0.1 %)	0 (0 %)	1 (0.2 %)	
Other	2 (0.1 %)	2 (0.1 %)	0 (0 %)	
Not transferred (admitted from home)	1746 (98 %)	1321 (98 %)	425 (98 %)	
**Elective procedure**	1728 (97 %)	1314 (98 %)	414 (96 %)	0.050
**American Society of Anesthesiologists (ASA) Classification**				0.26
1	4 (0.2 %)	2 (0.1 %)	2 (0.5 %)	
2	507 (29 %)	393 (29 %)	114 (26 %)	
3	1232 (69 %)	927 (69 %)	305 (71 %)	
4	35 (2.0 %)	24 (1.8 %)	11 (2.5 %)	
**Diabetes**	271 (15 %)	200 (15 %)	71 (16 %)	0.43
**Smoker**	114 (6.4 %)	107 (7.9 %)	7 (1.6 %)	**<0.001**
**Dyspnea on admission**	72 (4.0 %)	51 (3.8 %)	21 (4.9 %)	0.33
**Chronic obstructive pulmonary disease (COPD)**	58 (3.3 %)	47 (3.5 %)	11 (2.5 %)	0.34
**Congestive heart failure**	1 (<0.1 %)	1 (<0.1 %)	0 (0 %)	>0.99
**Hypertension**	733 (41 %)	563 (42 %)	170 (39 %)	0.36
**Concomittant cancer**	1 (<0.1 %)	1 (<0.1 %)	0 (0 %)	>0.99
**Steroid use**	43 (2.4 %)	31 (2.3 %)	12 (2.8 %)	0.58
**Functional status**				**0.046**
Independent	1635 (92 %)	1246 (93 %)	389 (90 %)	
Partially Dependent	122 (6.9 %)	82 (6.1 %)	40 (9.3 %)	
Totally Dependent	21 (1.2 %)	18 (1.3 %)	3 (0.7 %)	
**Bleeding disorder**	28 (1.6 %)	21 (1.6 %)	7 (1.6 %)	0.93

### 2.4 Statistical analysis

Continuous data are presented in median and interquartile ranges (IQR), while categorical data are presented in frequencies and proportions. A 2:1 propensity score-based matching protocol was employed to address confounders. Using the “optimal” matching approach, patients were matched based on the following variables: age, sex, race, BMI, emergency vs. elective setting, American society of anesthesiology (ASA) class, functional status, smoking status, dyspnea, diabetes, congestive heart failure, hypertension, chronic obstructive pulmonary disease, chronic steroid use, and time from admission to surgery.

**Fig. 1:**
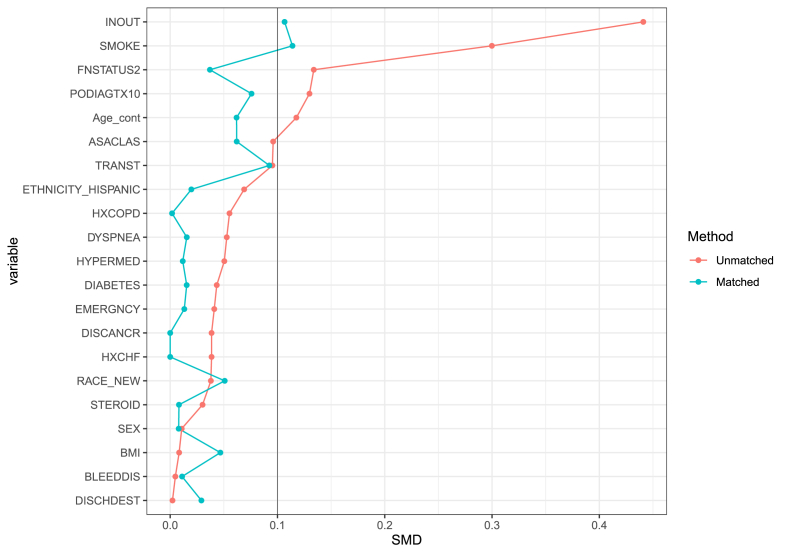
Love plots demonstrating the performance of the matching process. Each point represents the standardized mean difference of a covariate between our two groups, before and after adjustment.

Balance diagnostics for baseline clinical characteristics were evaluated before and after matching using standardized mean differences and Love plots (Fig. 1). Following matching, events were compared using Pearson's Chi-square, Fisher's Exact, and Man-Whitney U tests as appropriate. Statistical significance evaluated at p ≤ 0.05 for two-tailed tests. Statistical analysis was performed using R statistical software (version 4.3.1)

## 3 Results

### 3.1 Baseline characteristics

In total, 1778 patients undergoing asleep (n = 1346; 75.3 %) and awake DBS procedures (n = 432; 24.7 %) were identified in the NSQIP registry (Table 1). The median age among included was 68.0 (IQR: 61.0–73.0), with most being males (n = 1162; 65 %). The majority of patients undergoing DBS procedures were White (n = 1698; 96 %), followed by Black (n = 31; 1.7 %), and Asian (n = 49; 2.8 %). The indications for DBS treatment were Parkinson's disease (n = 1303; 73 %) and essential tremor (n = 475; 27 %).

Patients undergoing asleep DBS were slightly younger as compared to those who underwent awake DBS (67 vs. 68; p = 0.041). There were no significant differences in the distribution of races among anesthesia modalities (p = 0.80). Patients undergoing DBS for Parkinson's disease were slightly underrepresented in the asleep as compared to the awake group (72 % vs. 78 %; p = 0.021), while those with essential tremor were overrepresented in the asleep as compared to awake group (28 % vs. 22 %; p = 0.021).

There was a significantly higher percentage of smokers undergoing asleep DBS procedure (7.9 % vs. 1.6 %; p < 0.001). Also, a higher proportion of totally functionally dependent patients underwent asleep as opposed to awake DBS (1.3 % vs. 0.7 %; p = 0.046). However, this was the opposite for partially dependent patients (6.1 % vs. 9.3 %; p = 0.046).

Other parameters, such as sex, BMI, ethnicity, ASA classification, and specific comorbidities including diabetes, hypertension, and dyspnea, were represented equally in both groups (p < 0.05; Table 1).

### 3.2 Matched analysis comparing 30-day adverse events of asleep versus awake DBS

Propensity score matching (2:1) was performed to calibrate the cohorts with respect to the inherent baseline differences. After matching, no significant difference between the awake and asleep groups were detected across any of the baseline variables (Fig. 1; p ≥ 0.05). In the matched cohort, there were no differences in the 30-day risk of complications across both groups (2.3 % vs. 0.7 %, p = 0.062; Table 2). Moreover, there was no difference in the 30-day rate of readmission (3.5 % vs. 3.5 %; p = 0.96) and reoperation (1.4 % vs. 0.9 %; p = 0.48). Similarly, non-home discharge occurred to similar extents in both groups (3.5 % vs. 3.0 %; p = 0.63). Finally, the median length of hospital stay did not significantly differ between groups (0 vs. 0 days; p = 0.23).

**Table 2:** Thirty-day outcomes following the procedure in the post-propensity score matching cohort.

30-day outcomes	Overall, N = 1226	General, N = 794	Local, N = 432	p-value
**Duration of procedure (mins)**	47.0 (31.0, 133.0)	49.0 (35.0, 127.5)	40.0 (27.0, 144.0)	**<0.001**
**Total length of hospital stay**	0.0 (0.0, 1.0)	0.0 (0.0, 1.0)	0.0 (0.0, 1.0)	0.23
**Discharge disposition**				0.63
Home	1185 (97 %)	766 (96 %)	419 (97 %)	
Non-Home	41 (3.3 %)	28 (3.5 %)	13 (3.0 %)	
**Surgical site infection**	8 (0.7 %)	7 (0.9 %)	1 (0.2 %)	0.27
**Unplanned intubation**	1 (<0.1 %)	0 (0 %)	1 (0.2 %)	0.35
**Urinary tract infections**	3 (0.2 %)	2 (0.3 %)	1 (0.2 %)	>0.99
**Stroke**	1 (<0.1 %)	1 (0.1 %)	0 (0 %)	>0.99
**Myocardial infarction**	2 (0.2 %)	2 (0.3 %)	0 (0 %)	0.54
**Any complication**	21 (1.7 %)	18 (2.3 %)	3 (0.7 %)	0.062
**Unplanned 30-day readmission**	43 (3.5 %)	28 (3.5 %)	15 (3.5 %)	0.96
**Unplanned 30-day reoperation**	15 (1.2 %)	11 (1.4 %)	4 (0.9 %)	0.59

## 4 Discussion

DBS has evolved into a pivotal intervention for various neurological disorders. However, the ideal anesthesia modality during DBS electrode placement for ensuring the highest efficacy and least side effects remains debatable. This study aimed to study the potential risk factors of short-term adverse events associated with the use of awake or asleep techniques.

Our findings highlight inherent baseline differences among patients undergoing asleep versus awake DBS. Specifically, patients in the asleep group were slightly younger (p = 0.041), had a higher prevalence of Parkinson's disease compared to essential tremor (p = 0.021), and were more likely to have their procedures performed on an outpatient basis (p < 0.001). Additionally, smokers were significantly less represented in the awake group (p < 0.001), and awake patients had a higher rate of partial dependency in functional status (p = 0.046). These observations suggest distinct patterns regarding the current utilization of the two anesthesia modalities for DBS treatment.

Each of the two modalities come with distinct advantages and pitfalls (Tempelhoff, 2017). Although awake DBS is highly effective in ensuring precise electrode placement through continuous neurological monitoring and intraoperative testing, the procedure can be lengthy, causing discomfort for patients who are required to remain still on the operating room table (Gadot et al., 2023). However, with the advent of mini frames and frameless techniques, this issue is becoming less significant. Advances in brain imaging and intraoperative microelectrode recording now allow for precise electrode placement even under GA (Lee et al., 2022). In fact, in a trial comparing awake and asleep DBS, the latter was described as less burdensome by patients and on average 26 min shorter than awake DBS. It is important to note that while GA provides a secured airway, it may induce preprocedural anxiety in patients and comes with inherent risks associated with the anesthetic use (Tempelhoff, 2017).

Nonetheless, both techniques have repeatedly demonstrated similar short-as well as long-term outcomes. A meta-analysis of 14 studies including 1523 patients revealed similar clinical outcomes between asleep and awake DBS in terms of postprocedural improvement of symptoms as well as the rate of adverse events (Liu et al., 2019).

Similarly, another review concluded that the stereotactic targeting accuracy of lead placement obtained by either method is equally reliable as well as cost-effective (Wang et al., 2020). In addition, several randomized controlled trials have concluded non-inferiority of asleep DBS as compared to the traditional awake DBS in terms of operative complications as well as motor and functional postoperative outcomes (Holewijn et al., 2021; Engelhardt et al., 2021). While our findings only address short-term 30-day postoperative adverse events, they are nonetheless in keeping with the previously published evidence highlighting the state of equipoise between both approaches (Chen et al., 2017; Wang et al., 2020; Lim et al., 2024).

However, the fact that evidence has consistently pointed towards an equivalence between these two alternatives does not preclude the notion that one of the procedures may be better suited for a specific group of patients. Factors such as anxiety, cognitive impairment, or difficulties with intraoperative cooperation may favor an asleep approach, whereas patients prioritizing intraoperative testing for optimal lead placement may benefit more from the awake method. Future studies should aim to identify patient-specific predictors of optimal outcomes to support a more individualized, precision-guided approach to anesthesia selection in DBS.

### 4.1 Limitations

This study is subject to the limitations inherent to retrospective and registry-based studies. The data may suffer from potential data entry inaccuracies or underreporting. It is important to acknowledge that all patients ultimately undergo general anesthesia, as even those receiving awake DBS electrode placement are typically anesthetized for subsequent neurostimulator implantation. In many cases, this second stage occurs during the same operative session, which may attenuate differences in anesthesia-related risk profiles between the groups and represents a limitation to the binary asleep-vs-awake comparison. Additionally, this study was only able to focus on short-term outcomes, as long-term outcome data was absent. Similarly, patient-reported health-related quality of life outcomes which are crucial components in determining the efficacy and usability of a procedure are not provided by the NSQIP. While the potential cost-effectiveness advantages of each of the approaches may be reflected by discharge disposition, length of hospital stay, safety profiles, and reimbursement patterns, the lack of granularity of the database prevented a formal cost-effectivity analysis from being performed. For such quality assurance databased, it is also difficult to define data quality or to check potentially faulty data. Finally, it is important to note that most available results including ours are physician-reported, while patient-reported outcomes remain poorly studied. This gap in research may obscure potential underlying differences between the two approaches.

## 5 Conclusion

In this matched analysis using data from a prospective multicenter database of U.S. hospitals, asleep and awake DBS demonstrated comparable 30-day outcomes. No significant differences were observed in complication rates, readmissions, reoperations, discharge disposition, or length of hospital stay. These findings support clinical equipoise between the two approaches and underscore the importance of tailoring the choice of technique to individual patient characteristics and preferences.

## Declaration of competing interest

The authors declare that they have no known competing financial interests or personal relationships that could have appeared to influence the work reported in this paper.
